# Effect of *Centratherum anthelminticum* seeds on Glycated hemoglobin, Cardiovascular disease risks and dyslipidemia in Type-2 diabetic patients in Karachi: A non-blind randomized controlled trial

**DOI:** 10.12669/pjms.41.1.8496

**Published:** 2025-01

**Authors:** Hina Akram Mudassir, Syed Muhammed Talha Arshad, Munazzah Naheed, Sadia Anwer

**Affiliations:** 1 Hina Akram Mudassir Assistant Professor, Department of Biochemistry, Biochemistry, Federal Urdu University of Arts, Science & Technology, Karachi, Pakistan; 2 Syed Muhammed Talha Arshad Senior Medical Officer, HOD Diabetic Clinic, Sindh Government Hospital New Karachi, Karachi, Pakistan; 3 Munazzah Naheed Research Student, Biochemistry, Federal Urdu University of Arts, Science & Technology, Karachi, Pakistan; 4 Sadia Anwer Research Student, Biochemistry, Federal Urdu University of Arts, Science & Technology, Karachi, Pakistan

**Keywords:** *Centratherum anthelminticum* seeds, Fasting blood glucose, HbA1c, Diabetes

## Abstract

**Objective:**

To explore the effect of *Centratherum antheminticum* seeds powder {*Cap*_CASP_ 500 mg} capsule in diabetes Type-2 (T2DM) patients in Karachi.

**Methods:**

A randomized selection of 40 T2DM patients from Sindh Government Hospital New Karachi with their consents was done for a non-blinded controlled trial from October to December 2019 and divided into P_Group_ (Positive Control, metformin 500 mg) & T_Group_ (Test, *Cap*_CASP_ + was also included, using the same dosage of CapCASP on twenty healthy volunteers. The data were analyzed using an online graph pad student’s t-test and a one-way ANOVA (SPSS version 24) metformin 500mg each). Both groups had (n = 20). C_Group_ (healthy control), was also included with twenty healthy volunteers with same dose of *Cap*_CASP_. The biochemical tests (lipid profile, HbA1c, fasting blood glucose) of both groups were done in biochemistry lab Federal Urdu University of Arts, Science & Technology and results were analyzed by one-way ANOVA (SPSS version 24) and online graph pad student´s t-test

**Results:**

CASP showed potent (*in-vitro*) anti-diabetic activity (72.81%) than standard acarbose (standard drug). Most of *in-vivo* parameters in T_Group_ showed significant improvements. Body weight (*p*<0.01) & (*p*<0.05), HbA1c & fasting blood glucose (*p*<0.0001), coronary risk index (CRI), atherogenic index (A Indx) (*p*<0.05, *p*<0.0001) & atherogenic dyslipidemia ratio (A-DLR) (*p*<0.0001 & *p*<0.01) were reduced after six and twelve weeks. Reduction in serum cholesterol and triglycerides (*p*< 0.05 & *p*< 0.01) after six as well as (*p*< 0.01) after twelve weeks. Very low density lipoprotein reduced after both (*p*< 0.05) six and (*p*< 0.0001) twelve weeks. High density lipoprotein increased (*p*< 0.01) & low density lipoprotein decreased (*p*< 0.05) after twelve weeks.

**Conclusion:**

When given standard care to T2D patients, the CASP intervention demonstrated both anti-diabetic and anti-lipidemic effects.

## INTRODUCTION

Type-2 Diabetes is related to elevated blood glucose levels that leads to intricate metabolic uncertainty (↑ blood triglycerides hinder the insulin availability to cells,) expressed as hyperglycemia and insulin resistance.^1^ International Diabetes Federation already declared that in 2022, 26.7% (about 33 million) Pakistani population developed diabetes.^2^ Although China and India competed to reach half a billion by 2035 and 61.5 million respectively.^3^ The glycation of hemoglobin (Hb) predicts insulin resistance state of hyperglycemia and persistence of this process generate Reactive Oxygen Species (ROS) that alters red blood cells membrane as well as blood flow.^4^ Co-morbidities include Dyslipidemia and cardiovascular diseases that follow sustained Type-2 diabetes with irregular lipid/cholesterol markers in blood that lead to atherosclerosis and coronary artery disease development in future.^5^

Herbal induction alone or in combination (complementary) medicine provides a new dimension to cure multiple disorders and about 72.8% diabetic patients use dietary and herbal supplements.^6^ Centratherum anthelminticum, also known as kali zeeri in Pakistan, is a plant belonging to the Asteraceae family. Its ethanolic extract has been shown to enhance insulin secretion, lipid biomarkers^7^, reduce oxidative stress, and normalize HbA1c glycation.^8^ In an animal study, its seed oil (hexane fraction) recently demonstrated improvement in kidney problems connected to hyperglycemia.^9^

Due to anti-diabetic and anti-hyperlipidemic potential of *C. antheminticum* seeds, it can be a safe choice than available synthetic drugs.^10^ Because metformin regularly advised as base-line treatment and researchers reported its side effects (biochemical alterations) in diabetic patients.^11^ The high usage of anti-diabetic herbs is due to phytochemicals^12^, including Pakistan where awareness of ethno-medicine is common.^2^

This part of study was done alongside of reported study^13^, to investigate anti-diabetic effects of C. anthelminticum seeds powder capsule (CapCASP) on HbA1c, fasting blood glucose, and lipid biomarkers in Type-2 diabetic patients who are on metformin. Additionally, the trial aimed to examine the potential synergies between this herbal intervention and conventional therapy.

## METHODS

An unblinded randomized controlled trial with sixty participants (n =60) between the ages of 40 and 60 who had given their previous consent was carried out from October to December 2019. The 250 mg dose of CASP was chosen based on earlier research.^10^ Twenty healthy controls (n = 20) and forty Type-2 diabetic patients (n = 40) from Sindh Government Hospital New Karachi were included in the sixty subjects.

### Ethical approval:

Federal Urdu University of Arts, Science & Technology Research Ethics Committee and Hospital Review Board approved this work (Certificate Reference # 2032, date: 01-03-2018) and (Certificate # 560, date: 30-03-2018).

**Fig.1:**
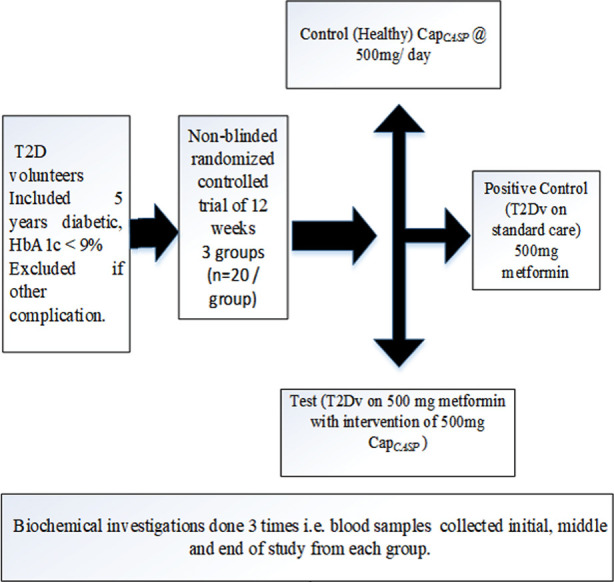
Study Design.

During this study *Centratherum anthelminticum* seeds were bought from market followed cleansing then grinded into fine powder and encapsulated (Courtesy of Maktum Homeo Pharma) as *Cap*_CASP_ (250mg). Three groups were randomly assigned to the participants. Several strategies were used to minimize the possibility of bias, including blinded data analysis by independent researchers, objective biochemical parameters (HbA1c, lipid profile) being used as primary outcome measures, and randomly assigning participants to treatment groups. Inclusion criteria {T2D participants, male (n= 28) and female (n= 12)} both with diabetic history of five years, HbA1c between 7% to 9% with no other complaints (Table-I). All were dispersed into three groups (Fig.1) trailed their treatments protocol (daily intake of 2 *Cap*_CASP_ (250mg) one before breakfast and one after dinner for 12 weeks) and in contact with Diabetologist in diabetes care unit of the hospital. Their body weight (BW) and fasting blood glucose (FBG) were supervised weekly and blood samples were drawn three times (first day, after 6-12 week) for biochemical investigations at biochemistry Lab FUUAST respectively. Serum cholesterol (TC)^14^, triglycerides (TG)^15^, high density lipoprotein (HDL-c)^16^, low density lipoprotein (LDL-c)^17^ was done by using Randox kits, UK. Very low density lipoprotein (VLDL-c) was estimated through the standard formula^18^, VLDL-c = tg/5. Coronary risk index (CRI) was estimated through standard formula^18^,

**Table-I:** Baseline characteristics of selected subjects.

Characteristics	C _Group_	P _Group_	T _Group_
Age (years)	44 ±10	45.3±8.7	49.7 ±9.4
Male (n)	11	15	14
Female (n)	09	05	06
Body mass(kg)	55 ±4.0	78 ±12.24	75 ±12.25
Fasting blood glucose (mg/dl)	88.5 ±8.96	193.7 ±30.89	198 ±57.87
HbA1c (mg/dl)	5.18 ±0.47	8.3 ±1.18	8.4 ±1.43
BMI (kg/m^2^)	20.8±2.1	26±3.04	25±3.3

values are mentioned as means ± SD, n=20 in each group.

CRI = TG / HDL-c

Other ratios like Atherogenic dyslipidemia ratio (ADR) and Atherogenic index (AI) were estimated through the following formula^19^ respectively,

ADR = {log (TG)/ HDL-c}

AI = LDL-c / HDL-c

### In-vitro Anti-diabetic activity of Centratherum anthelminticum seeds powder:

The activity of powdered *Centratherum anthelminticum* seeds was tested against α-glucosidase. Using phosphate buffer (pH 6.8), the total temperature of the experimental method was maintained at 37°C. After dissolving the α-glucosidase enzyme in phosphate buffer (0.02 U/well) at 25 ºC, the test substance was added and incubated for 15 minutes. Subsequently, 0.7 mM of p-nitrophenyl-α-D-glucopyranoside (substrate) was added, and absorbance was measured for 30 minutes at 400 nm. DMSO (7%), a positive control, was employed. Standard medication acarbose was utilised as a competitive medicine.^20^ % Applying the formula above, inhibition was determined.

*100 - (OD test well/OD control) × 100 = %Inhibition*

### Statistical Analysis:

Results are expressed as mean ±SD (standard deviation) and resolute as significant at *p<* 0.05, *p<* 0.01, *p<* 0.0001 when analyzed by one-way ANOVA (SPSS version 24) and online graph pad student´s t-test.

## RESULTS

### Anti-diabetic activity of IC_50_ Centratherum anthelminticum seeds (in-vitro):

The α- glucosidase assay of *C. anthelminticum* seeds powder showed good anti-diabetic activity i.e. IC_50_ value i.e. 102.68µg ±1.43 (72.81%) as compared to standard Acarbose, IC_50_ value 377.71µg ±1.34.

### Baseline Characteristics:

There were 60 participants in the pilot trial (20 healthy, 40 T2D). Table-I primarily displays the demographic values that were comparable between the diabetic groups.

### Cap_CASP_ effect on body weight, fasting blood glucose and HbA1c:

**Fig.2:**
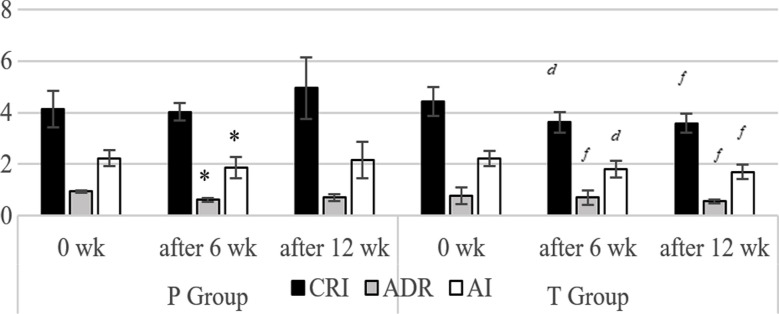
Effect of Cap_CASP_ on CRI, ADR & AI ratios in diabetic volunteers. All bars are mean ± SD n=20, *^d^p<* 0.05*, ^e^p<* 0.01*, and ^f^p<* 0.0001, when comparing with P_Group_ *^#^p<*0.05, ^*^ p<0.01 *& ^+^p<*0.0001 when individual group compared with initial (1^st^ day) record

*Cap*_CASP_ after six weeks reduced body weight values (*p*<0.01) significantly in all groups than their initial records (Table-II). In diabetic groups the T_Group_ showed significant reduction (*p*<0.01) than P_Group_ after six weeks though C_Group_ showed more reduction (*p*<0.0001) with in the same time gap. Likewise, after 12 weeks, body weight reduced in T_Group_ (*p*<0.05) and C_Group_ (*p*<0.01) respectively (Table-II). Body weight also reduced (*p*<0.05) in T_Group_ after 12 weeks than its first records (Table-II). Fasting blood glucose values also improved in all groups, reduced significantly (*p*<0.0001) after six and 12 weeks than their initial records. T_Group_ showed significant reduction in fasting blood glucose and HbA1c (*p*<0.0001) after six and 12 weeks than P_Group_ (Table-II). HbA1c values significantly reduced (*p*<0.05) after six and 12 weeks in T and C_Group_ when compared to their initial records (Fig.2) even C_Group_ showed better reduction (Fig.2).

**Table-II:** Effect of Cap_CASP_ on body weight (BW), fasting blood glucose (FBG) and HbA1c in diabetic and healthy volunteers.

Groups	Body weight (kg)			Fasting blood glucose (mg/dl)			HbA1c (mg/dl)		

	1^st^ day	After 6 weeks	After 12 weeks	1^st^ day	After 6 weeks	After 12 weeks	1^st^ day	After 6 weeks	After 12 weeks
C _Group_	54.5 ±4.0	51.33 ±1.21^f*^	52.25 ±2.9^e^	88.50 ±8.96	75.16 ±13.07*^+^	79.5 ±10.13^#+^	5.18 ±0.47	4.7 ±0.07^+^	5 ±0.33^+^
P _Group_	78 ±12.24	77.66 ±11.57^*^	79.33 ±12	193.66 ±30.89	188.90 ±23.34^+^	186.33 ±36.1	8.3 ±1.18	8.23 ±1.17	8.4 ±1.5
T _Group_	75.3 ±12.07	71.25 ±14.26^e*^	73 ±10^d#^	198.00 ±57.87	121.30 ±35.24^+f^	114.25 ±28.81^+f^	8.4 ±1.43	7.09 ±0.09^#f^	6.79 ±0.67^#f^

values are mentioned as mean ± SD,

d p< 0.05,

e p< 0.01, and

f p< 0.0001, when comparing with P_Group_

# p<0.05,

* p <0.01 &

+ p<0.0001 when individual group compared with initial (1^st^ day) record.

### Serum lipids and lipid ratios {Coronary risk index(CRI), Atherogenic index (AI), Atherogenic dyslipidemia ratio (ADR)}:

Serum TC and TG levels in C_Group_ decreased significantly (*p*< 0.05 & *p*< 0.0001) after six weeks and (*p*< 0.01 & *p*< 0.05) after 12 weeks than their first records whereas serum VLDL level was reduced (*p*< 0.01) after six and 12 weeks in C_Group_ and T_Group_ in comparison to initial records .Serum VLDL in C_Group_ was decreased (*p*< 0.01) after six and 12 weeks than its initial values (Table-III). In P_Group_ a significant drop in serum TC (*p*< 0.0001), TG and LDL (*p*< 0.01) after six weeks than first records.. The serum HDL values were significantly increased (*p*<0.01and *p*< 0.0001) in T_Group_ after six and twelve weeks than its first record whereas C_Group_ and P_Group_ showed increased serum HDL level (*p*< 0.01) after six weeks than their first records (Table-III). T_Group_ showed significant reduction in serum TC and TG (*p*< 0.01) after 12 weeks while after 6 weeks tc showed significant reduction (*p*< 0.05) than P_Group_. Serum VLDL values was also significantly reduced in T_Group_ (*p*< 0.05) after six weeks, (*p*< 0.01 & *p*< 0.0001) after 12 weeks while serum LDL was decrease (*p*< 0.05) significantly after 12 weeks when compared to P_Group_.

**Table-III:** Effect of Cap_CASP_ on Serum lipids in diabetic and healthy volunteers.

Serum lipids (mg/dl)	C _Group_			P _Group_			T _Group_		

	1^st^ day	After 6 weeks	After 12 weeks	1^st^ week	After 6 weeks	After 12 weeks	1^st^ week	After 6 weeks	After 12 weeks
TC	147±24	141±29^# +^	130±16*	196±33	163±12^+^	190±46	176±32	150±16^d*^	149±20 *
TG	108±26	71±11^#+^	69±11^#^	189±50	155±19*	202±41	196±48	185±44	156±27 *
LDL	92.16±10	92.3±14	96.03±2.2	86.3±22.4	76.55±10*	83.5±19	83±13	76.6±10*	71±13*
HDL	40±5.7	40±7.1*	41.6±3.2	37±5.1	40±1.1*	38.7±4	38±2	41±2*	41±2.2^e+^
VLDL	22±5.1	14±2.5*	13.6±2*	40.86±8.7	40.4±10.3	40.4±10	40±16	32.63±4.6^d*^	24.24±3.2^f*^

Values are mentioned as mean ± SD n=20,

d p< 0.05,

e p< 0.01, and

f p< 0.0001, when comparing with P_Group_

# p<0.05,

* p<0.01 &

+ p<0.0001 when individual group compared with initial (1^st^ day) record.

The serum HDL values showed a significant increase (*p*< 0.01) in T_Group_ after 12 weeks when compared to P_Group_. Serum TC, LDL and VLDL in T_Group_ were significantly reduced (*p*< 0.01) after six and 12 weeks whereas serum TG showed significant (*p*< 0.01) reduction after 12 weeks than their initial value. Serum HDL in T_Group_ showed significant increase (*p*< 0.01) after six and (*p*< 0.0001) after 12 weeks than its initial record (Table-III).

T_Group_ showed a significant fall in ADR (*p*<0.0001), CRI and AI (*p*<0.01) after six weeks whereas same three ratios reduced significantly (*p*<0.0001) after 12 weeks than P_Group_ (Fig.2). ADR and AI ratios in P_Group_ showed significant decrease (*p*< 0.01) after six weeks whereas T_Group_ showed significant decrease *p*<0.01) in ADR, (*p*<0.0001) in CRI and AI ratios after 6 and 12 weeks than their first records (Fig.2).

## DISCUSSION

In this work, the *in-vitro* study of *CASP* showed good anti-diabetic activity against standard drug, acarbose confirming its previously reported glycation resistance feature, which has manifested as *in-vivo* findings in this research. The improved HbA1c and fasting blood glucose levels in diabetic test (T_group_) than diabetic control (P_group_) (Fig.2, Table-III) after 12 weeks^10^, confirming the possible phytochemicals role (flavonoids, polyphenols, alkaloids) in gut.^8^ In the past, it was reported that the major antidiabetic ingredients flavonoids (quercetin), alkaloids (berberine), terpenes (thymoquinone), and phenylpropanoids (chlorogenic acid) inhibited intestinal glucosidase, activated amylase, and increased the production of insulin, among other effects, all directed towards glucose utilisation, particularly in muscles and adipocytes that brought about the rejuvenation of beta cells.^10^,^21^ After six weeks, metformin improved glycaemic control and lowered the risk of CVD (Table II). However, when combined with CASP, better lipid content suggested the potential of lipoprotein lipase activation^22^, which boosted the breakdown of LDL as previously mentioned.^23^ Metformin (anti- diabetic drug derived from the plant *Galegine officinalis*) recently (dose ≤ 500 mg) showed a positive relationship with herbal induction in improving hyperglycemia in Type-2 diabetes^24^ that also endorsed the present results. In Tables-II and III, CASP demonstrate intervention was also successful in reducing body weight increase, lipid profile (TC, TG, LDL, VLDL) values, and lipid ratios in T2D patients compared to the metformin-treated T2D group. These findings appear to be consistent with earlier research.^7^,^8^,^25^

Conveniently, there is an adequate research available on *C. anthelminticum* seeds that showed lipid and glucose lowering effects in both diabetic^8^ and high cholesterol models.^7^ In fact, anti-obesity property of *C. anthelminticum*^23^ has also been reported. Captivatingly alike drop in the values of lipid profile of healthy subjects (C_group_) endorsed the hypolipidemic property of CASP (Table-III). CASP might improve cholesterol levels by locking HMG-CoA reductase (↓cholesterol synthesis & ↑ LDL degradation),^9^ and it was also exhibited in early detective markers including ADR, AI and CRI ratios of diabetic CVD risk factor^2^ showed improved values in test subjects (Fig.2).

In practice, anti-diabetic and lipid lowering drugs single or in amalgamation presented challenges in the management of macrovascular problems.^22^ Therefore, CASP interventions (presence of oxidation scavenger and enzyme inhibitors like α-amylase and α-glucosidase) can be a multi-tasking T2D treatment along with routine medicine like metformin with no obvious side effects that ensure the possible role of *C. anthelminticum* seeds to cure secondary challenges in future.

### Limitations:

The results of this work do not reflect the complete population and cannot be generalised to all Type-2 diabetes patients utilising medicinal herbs due to the limited number of T2D patients (it is a pilot study).

## CONCLUSION

*C. anthelminticum* seeds provided a potential future antidiabetic medicine, also facilitated metformin performance without adverse effects. This interaction of natural elicitors with metformin chemistry will be our next scheme in future research.

### Authors’ Contributions:

**HAM:** Conceived and designed the study.

**MN** and **SA:** Collected the data and performed laboratory tests and statistical analysis.

**HAM** and **SMTA:** Manuscript writing and responsible for the integrity of the study.

All authors have read and approved the final manuscript.
